# Point-of-Care Ultrasound Curriculum for Internal Medicine Residents During the COVID-19 Era: A Pilot Study

**DOI:** 10.7759/cureus.25944

**Published:** 2022-06-14

**Authors:** Sarbjot Grewal, Arielle Houston, John Bacon, Earvin Balderama, Mohammed G Elhassan

**Affiliations:** 1 Internal Medicine, Saint Agnes Medical Center, Fresno, USA; 2 Statistics, California State University, Fresno, Fresno, USA

**Keywords:** graduate medical education (gme), gme, education, medical residency, ultrasound (u/s), pocus (point of care ultrasound)

## Abstract

Introduction: The use of point-of-care ultrasound (POCUS) by internal medicine physicians and residents is increasing. We present the results of a pilot study to implement a POCUS curriculum that was interrupted by the Coronavirus Disease 2019 (COVID-19) pandemic at an internal medicine residency program at a community hospital. The purpose of this study is to inquire about the attitude and interest of our medical residents in POCUS. Additionally, we also plan to examine whether a curriculum that lacks some practical aspects due to COVID-19 restrictions can still improve the residents’ confidence in recognizing common POCUS applications and improve image interpretation skills.

Methods: We conducted a prospective, pre-, and post-curriculum pilot study to examine the POCUS skills of categorical internal medicine residents in Post-Graduate Years (PGY) 1 through 3 at a community hospital. The two POCUS-related skills examined were self-reported confidence level in recognizing certain POCUS examination findings and POCUS image interpretation skills. Due to social distancing guidelines, we were unable to host hands-on sessions as originally planned, but residents did receive lectures via Zoom regarding POCUS training and also organ-specific diagnoses. Three primary outcomes were measured: (1) baseline difference in confidence level between interns (PGY-1) and senior residents (PGY-2 and 3) at the beginning of the curriculum, (2) improvement in POCUS confidence level before and after the curriculum considering interns and senior residents all together and also separately, and (3) improvement in image interpretation skills before and after the curriculum.

Results: Of 41 residents, 23 participants completed the pre- and post-curriculum test. Of the 23 participants, 12 participants were interns, and 11 were senior residents. Overall, interns showed a statistically significant improvement in the confidence level in almost all diagnoses except pulmonary embolism (p = 0.084). For image interpretation tests, significant improvement was found only in recognizing the two signs of pneumothorax: pleural line absent sliding (X^2^ = 4.00, p < 0.05) and the barcode sign (X^2^ = 6.13, p < 0.05). The pre-curriculum confidence level questionnaire included a question about residents’ interest in learning POCUS during residency. It showed that the vast majority of residents (21 residents [91%]) are either extremely or mostly interested in POCUS. Most of our residents (18 [78%]) did not have formal exposure to POCUS during medical school.

Conclusion: A POCUS curriculum that lacks hands-on workshops and longitudinal image saving and reviewing due to the COVID-19 pandemic restrictions did not improve the residents’ image interpretation skills, although the confidence levels of the interns statistically improved. After the pandemic, we plan to implement the full curriculum and examine whether it will improve the residents' image acquisition and interpretation skills.

## Introduction

Ultrasonography is a safe and effective type of imaging that has been utilized for decades by physicians to assist with diagnosis and treatment. Unlike other forms of imaging, ultrasound does not require ionizing radiation or strong magnetic fields to yield clinically useful imaging. It is a fast, cheap, and safe modality that lacks the downsides of cost and radiation exposure that afflicts other types of imaging. Ultrasound probes have significantly improved in quality and decreased in cost over the last 30 years, leading to the proliferation of high-quality and low-cost probes that have made ultrasonography increasingly ubiquitous. Clinicians and physicians-in-training will likely face an environment one day when the ultrasound probe is as ubiquitous as the stethoscope, and point-of-care ultrasound (POCUS) diagnosis of various illnesses might be the standard of care [1]. POCUS is an ultrasonography performed and interpreted by the clinician at the bedside [2]. Ultrasound is, perhaps, the most user-dependent imaging modality currently available [3], so adequate training in the acquisition and interpretation of obtained images is critical. This user-driven nature of the modality and its increasing prominence have caused graduate medical education programs to acknowledge the importance of early training for tomorrow’s clinicians. It has been incorporated into various training programs, especially in Emergency Medicine, Critical Care, Obstetrics, and Gynecology [4,5]. Internal medicine residences have not had a rate of adoption of the POCUS curriculum as high as these specialties, but an increasing number of residences has begun incorporating ultrasonography into their training.

In this article, we present the results of a pilot study to implement a POCUS curriculum that was interrupted by the Coronavirus Disease 2019 (COVID-19) pandemic at a small internal medicine residency program at a community hospital. The purpose of this study is to inquire about the attitude and interest of our medical residents in POCUS and to examine whether a curriculum that lacks some practical aspects due to COVID-19 restrictions can still improve the residents’ confidence in recognizing common POCUS applications and improve their image interpretation skills.

## Materials and methods

We conducted a prospective, pre-, and post-curriculum study to examine the POCUS skills of categorical internal medicine residents in Post-Graduate Years (PGY) 1 through 3 at a community hospital. The two POCUS-related skills examined were self-reported confidence level in recognizing certain POCUS examination findings and POCUS image interpretation skills. The study period was from July 1, 2020, through May 31, 2021, during the third academic year of this new residency program (established in 2018) and also during the lockdown period of the COVID-19 pandemic. Approval for the study was obtained from the Saint Joseph Mercy Health System Institutional Review Board (Approval No.: NHSR-22-1013).

Participating residents took the pre-curriculum confidence level survey and the image interpretation test prior to initiation of the curriculum. All internal medicine residents were encouraged to participate, but enrollment was strictly on a voluntary basis. Using a Likert scale from 1 to 5 (1 being not confident at all and 5 being extremely confident), eight questions examined the residents’ confidence levels in identifying common POCUS applications relevant to internal medicine. The image interpretation questions consisted of 18 multiple choice questions, created by the senior author who has a six-year of experience in POCUS in the inpatient setting, to examine whether residents can interpret POCUS images with short inpatient clinical scenarios. The residents received a protected 30-45-minute session to complete the exam. No outside materials were allowed, and participants were not allowed to discuss the questions with each other during the exam. The same survey and test were repeated at the end of the academic year.

The POCUS curriculum

After taking the initial survey and test, all residents participated in the POCUS curriculum that included monthly POCUS lectures done via Zoom, sharing and discussing POCUS cases using the WhatsApp application, and individual one-to-one mentoring during routine daily rounds of the two faculty hospitalists who were trained on POCUS. Each lecture, 30 minutes in length, focused on organ-specific findings, including slides and short videos demonstrating correct techniques of using ultrasound machines and organ-specific ultrasound findings as well. The faculty trained on POCUS used handheld devices (Butterfly iQ) for their one-to-one bedside demonstration during their routine rounds, and for some, they used images during the Zoom lectures.

Some parts of the curriculum were not conducted as originally planned due to social restrictions mandated by the pandemic. These included periodic (every four to six months) hands-on workshops utilizing healthy volunteers to train residents on image acquisition skills and recognizing normal patterns, classroom lectures with demonstrations of POCUS skills, and purchasing an online platform where residents can save their images to be reviewed at a later date by faculty. Therefore, a decision was made to conduct the study as a pilot study in preparation for a more comprehensive POCUS curriculum in the near future after the pandemic restrictions are eased.

Statistical analysis

Only the participants who took the survey and/or the image test before and after the curriculum were included for analysis. Three primary outcomes were measured: (1) baseline difference in confidence level between interns (PGY-1) and senior residents (PGY-2 and 3) at the beginning of the curriculum, using two-sided Wilcoxon rank-sum tests; (2) improvement in POCUS confidence level before and after the curriculum considering interns and senior residents all together and also separately, using one-sided Wilcoxon signed-rank tests; and (3) improvement in image interpretation skills before and after curriculum using McNemar’s chi-squared tests. For the Wilcoxon rank-sum and signed-rank tests, the test statistic is reported along with the p-value and the rank-biserial correlation as a measure of effect size. Secondary outcomes include questions about residents’ level of interest in learning POCUS during residency and the percentage of residents who had prior POCUS exposure before residency. The results of secondary outcomes were expressed in descriptive statistics. Only the senior author was able to identify respondents in order to be able to match the pre- and post-test results. All statistical analyses were done using R software version 4.1.2.

## Results

First primary outcome

**Figure 1:**
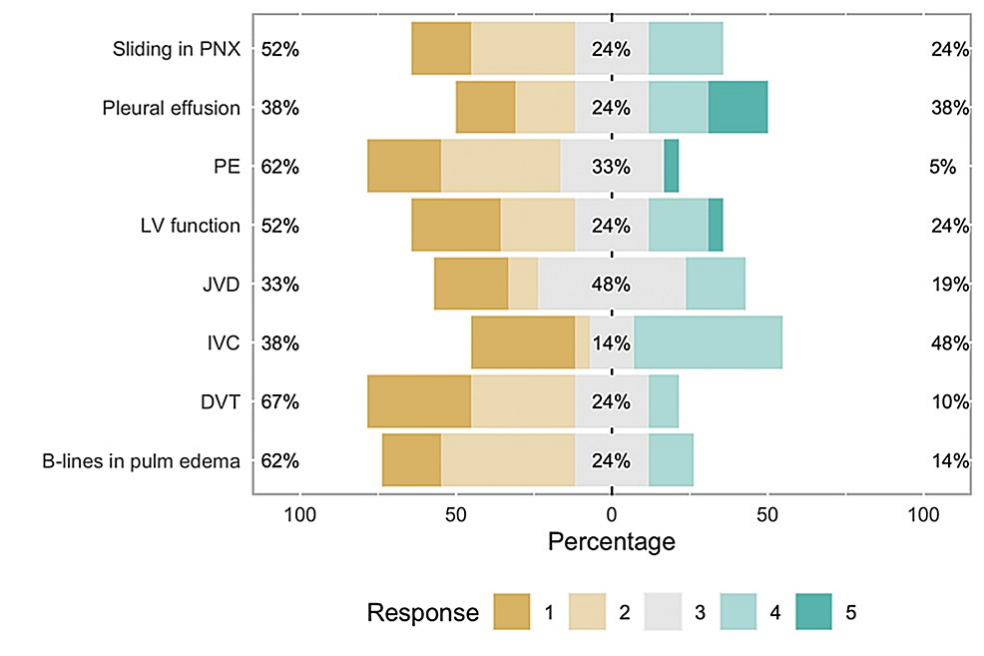
Bar graph showing pre-curriculum survey results This graph shows the majority of the residents do not feel comfortable diagnosing the above pathologies via the POCUS exam. PNX: Pneumothorax; PE: Pulmonary embolism; LV: Left ventricle; JVD: Jugular venous distention; IVC: Inferior vena cava; DVT: Deep vein thrombosis; POCUS: Point-of-care ultrasound.

**Figure 2:**
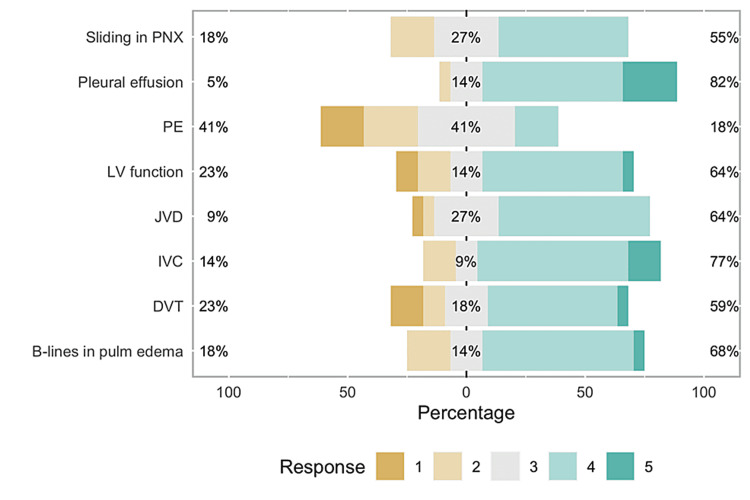
Bar graph representing post-curriculum survey This image shows that most residents felt comfortable using POCUS for diagnosing all diagnoses except PE and DVT mostly. PNX: Pneumothorax; PE: Pulmonary embolism; LV: Left ventricle; JVD: Jugular venous distention; IVC: Inferior vena cava; DVT: Deep vein thrombosis; POCUS: Point-of-care ultrasound.

Of 41 eligible participants, we had 23 participants (response rate 56%) who were able to complete the pre-curriculum and post-curriculum confidence level assessments. Of these 23 participants, 12 participants were interns and 11 were senior residents. As shown in Figure 1, senior residents appear to be more confident than interns in recognizing the following exams: B-lines in cardiogenic pulmonary edema (U = 90, r = 0.67, p < 0.01), pleural effusions (U = 97, r = 0.80, p < 0.01), grading left ventricular function (U = 99, r = 0.65, p < 0.01), inferior vena cava (IVC) diameter and collapsibility (U = 106, r = 0.77, p < 0.01), and jugular venous distention (JVD) (U = 98.5, r = 0.64, p < 0.01). There was no statistically significant difference found between the two groups in their baseline confidence for three skills: recognizing sliding pleural line in pneumothorax (U = 77, r = 0.43, p = 0.098), signs of large pulmonary embolism (U = 70, r = 0.17, p = 0.509), and recognizing deep venous thrombosis (DVT) (U = 64.5, r = 0.08, p = 0.782), depicted in Figure 2.

Second primary outcome

**Figure 3:**
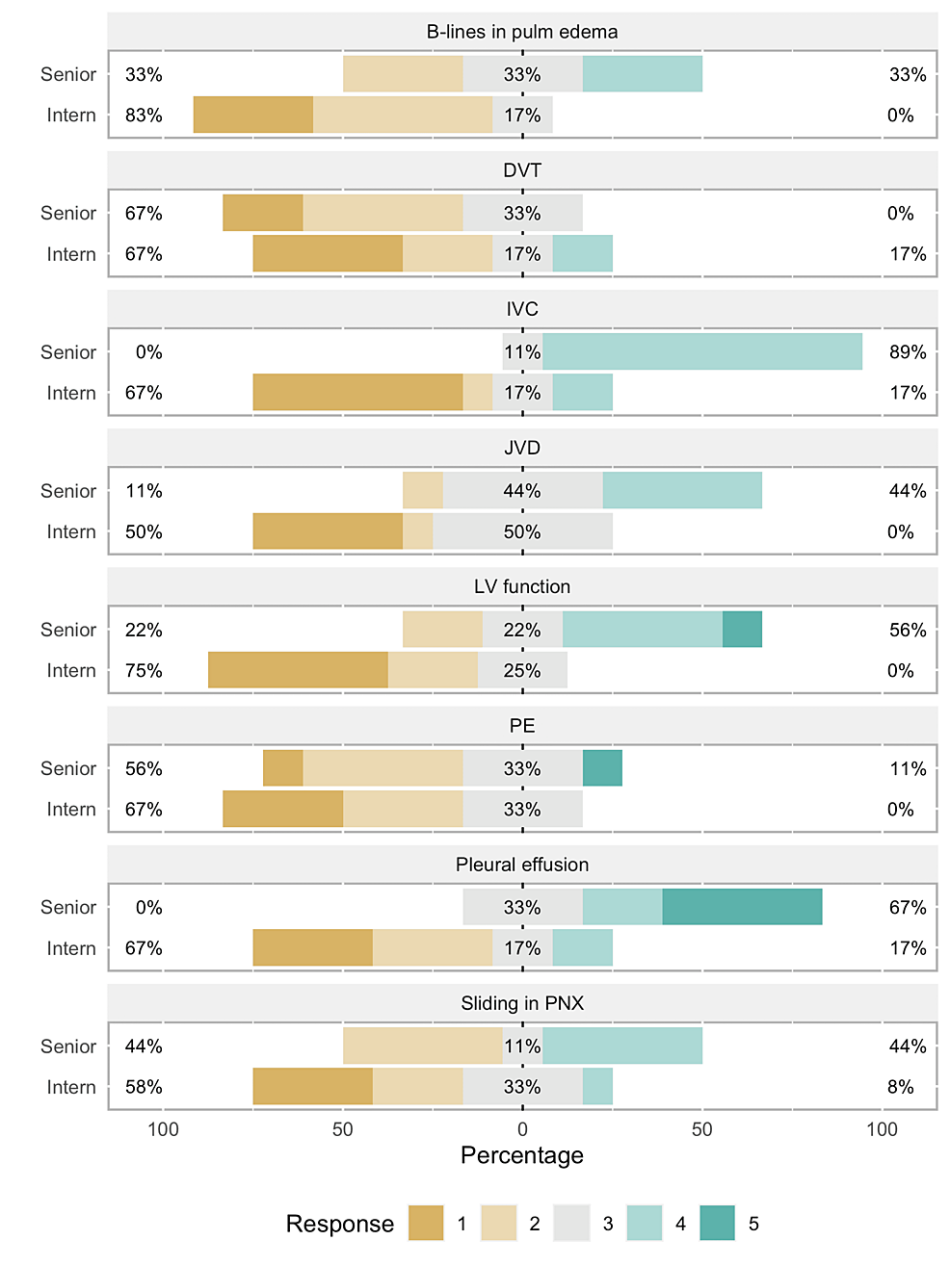
Bar graph showing pre-curriculum test results This image depicts that majority of the interns did not score well as compared to the senior residents. PNX: Pneumothorax; PE: Pulmonary embolism; LV: Left ventricle; JVD: Jugular venous distention; IVC: Inferior vena cava; DVT: Deep vein thrombosis.

**Figure 4:**
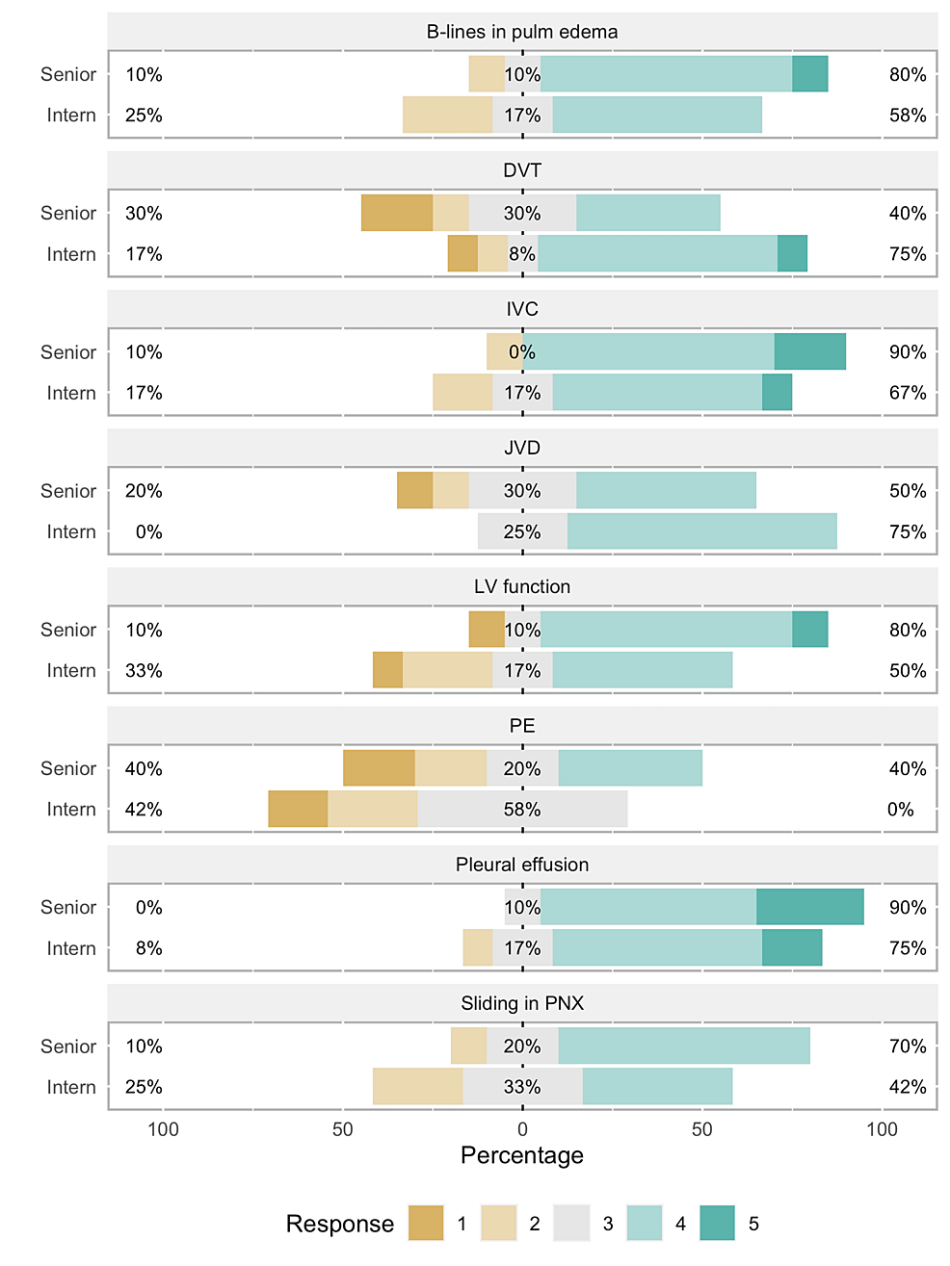
Bar graph representing post-curriculum test This image shows an improvement in scores especially for interns compared to senior residents, but the results were not statistically significant. PNX: Pneumothorax; PE: Pulmonary embolism; LV: Left ventricle; JVD: Jugular venous distention; IVC: Inferior vena cava; DVT: Deep vein thrombosis.

Interns showed a statistically significant improvement in confidence level in almost all POCUS skills: recognizing sliding pleural line in pneumothorax (T = 0, r = 1.00, p < 0.01), B-lines in cardiogenic pulmonary edema (T = 0, r = 1.00, p < 0.01), identifying pleural effusions (T = 0, r = 1.00, p < 0.01), grading left ventricular function (T = 8, r = 0.79, p < 0.01), IVC diameter and collapsibility (T = 0, r = 1.00, p < 0.01), JVD (T = 0, r = 1.00, p < 0.01), and finding DVT (T = 0, r = 1.00, p < 0.01). Figures 3, 4 compare the pre- and post-curriculum tests. Recognizing signs of large pulmonary embolism (T = 2, r = 0.73, p = 0.084) was not statistically significant at the 0.05 level but showed a large effect size in favor of improvement, owing to a majority of interns receiving the same score in both pre- and post-tests. Since the pre-curriculum confidence level of senior residents was already much higher than interns for most of the skills, there was not much room for substantial improvement.

Third primary outcome

Of the 14 participants who completed the baseline image interpretation test, only two did not complete the post-curriculum test. Twelve residents (all levels) were included in the analysis for the image interpretation test. Significant improvement was found only in recognizing the two signs of pneumothorax: pleural line absent sliding (X^2^ = 4.00, p < 0.05) and the barcode sign (X^2^ = 6.13, p < 0.05) (Figure 4).

Secondary outcomes

**Figure 5:**
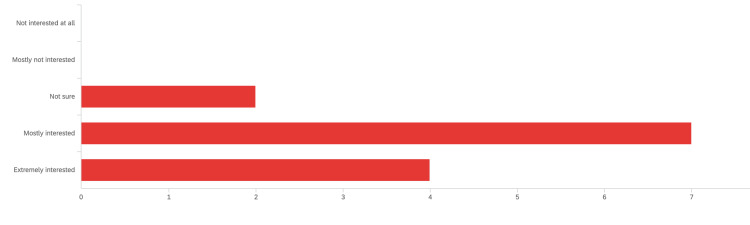
Response to the question: “Overall, how would you rate your interest to learn POCUS during residency?”

**Figure 6:**
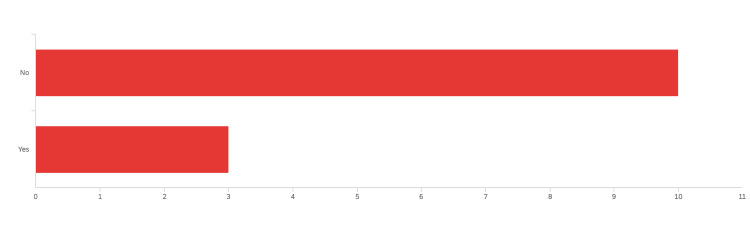
Response to the question: “Regarding bedside ultrasound or point-of-care ultrasound (POCUS): did you have any kind of formal courses/training/lectures about POCUS in medical school? If Yes, please mention what form of training (e.g., lectures, hands-on-experience, simulation, etc.).” POCUS: Point-of-care ultrasound.

The pre-curriculum confidence level questionnaire included a question about residents’ interest in learning POCUS during residency. Figure 5 shows that the vast majority of residents (21 residents [91%]) are either extremely or mostly interested in POCUS. Figure 6 shows most of our residents (18 [78%]) did not have formal exposure to POCUS during medical school.

## Discussion

Consistent with other studies, our residents seem to be interested in learning POCUS skills during residency. A survey done in the Netherlands in 2019 showed that 92% of the Internal Medicine residents (227 of 247 residents) believed that ultrasound is a useful skill, but about 40% of them had never performed an ultrasound due to a lack of formal guidance and supervision [6]. Multiple European societies have incorporated ultrasound skills as a mandatory requirement and are currently developing a core ultrasound curriculum for trainees [7,8].

Only three interns and two seniors among the respondents (five out of 18 respondents) said that they had some prior training and/or exposure to POCUS during medical school. Senior residents (PGY-2 and 3) in this program had informal exposure to some aspects of POCUS skills during the year that preceded the start of the curriculum and was in the form of casual use of POCUS during inpatient rounds by two faculty members. This might explain the statistical difference in confidence levels in most of the POCUS exams mentioned in the survey in favor of senior residents. There was more improvement in the confidence level noted with interns in comparison to their seniors likely due to the novice level associated with beginning one’s medical career in comparison to previously learned interpretations by their seniors.

We believe there were multiple reasons for which the current curriculum did not improve the resident’s skills in the image interpretation test. Due to the COVID-19 pandemic, hands-on workshops were not instituted as originally planned due to social distancing guidelines mandated by the hospital. A similar POCUS curriculum study at a small Internal Medicine Program included a total of nine-hour workshop that was spread over a few weeks along with longitudinal hands-on experience, which showed an improvement in the confidence survey scores and pre- and post-assessments as well [9]. We recognize that ultrasound is a user-dependent tool, and appropriate education and familiarity are of utmost importance during utilization. Another study in Canada confirms similar results showing statistically significant improvement in PGY-3 scores in comparison to PGY-1. Scores on the knowledge test improved based on the time spent in the curriculum, with the PGY-1 class scoring an average of 70.0% (21/30) and PGY-3 scoring 82.8% (24.9/30; p = 0.02) [10].

Also, the originally planned purchase of an image-saving platform to serve as an opportunity for residents to save their images in order to receive feedback from faculty, and due to financial restraints during the pandemic, the purchase was postponed. This was expected to improve the residents' image acquisition as well as image interpretation skills and even patient management as shown in other studies [9,11].

We plan to repeat the POCUS curriculum after the pandemic is over with the incorporation of hands-on workshops and an image acquisition platform. We can use the results of this pilot study and reach out to leadership and stakeholders to assist in reallocating resources to help purchase the intranet platform and complete the process that was delayed due to the pandemic. We would like to analyze the results of this pilot POCUS study with the one after the pandemic.

Our study has its limitations. The sample size is small despite the large interest in POCUS training, and this can affect generalizability. Because of the pandemic, residents were busy during inpatient and ICU rotations, and some were eventually pulled from their elective rotations. This might have shifted their attention and energy away from learning POCUS skills and participating in the study. We expect the periodic hands-on workshops to improve recruitment in future studies. As a community hospital program, the POCUS resources, including faculty trained in POCUS, are limited, and thus the residents’ exposure to POCUS outside of the curriculum was also limited. Lacking some aspects of the curriculum (e.g., the periodic hands-on training and image-saving platform) hindered the assessment of an important aspect of the POCUS curriculum: image acquisition skills.

## Conclusions

POCUS is gradually becoming an essential part of internal medicine training and can aid in diagnosing various medical conditions and may help change management. In our pilot study, we found that only first-year residents’ confidence level, but not image interpretation skills, improved with a curriculum that was interrupted by the pandemic and accordingly lacked some elements of a longitudinal curriculum such as periodic hands-on training and image-saving platform. We hope to reinstate this curriculum during the upcoming academic year and analyze the differences in results during the pandemic and post-pandemic training period.
